# Modeling ENSO impact on rice production in the Mekong River Delta

**DOI:** 10.1371/journal.pone.0223884

**Published:** 2019-10-22

**Authors:** Bui Tan Yen, Nguyen Huu Quyen, Trinh Hoang Duong, Duong Van Kham, T. S. Amjath-Babu, Leocadio Sebastian

**Affiliations:** 1 Soil and Fertilizer Research Institute, Hanoi, Vietnam; 2 Climate Research and Forecasting Division, Viet Nam Institute of Meteorology, Hydrology And Climate Change, Hanoi, Vietnam; 3 Agricultural Meteorology Division, Viet Nam Institute of Meteorology, Hydrology And Climate Change, Hanoi, Vietnam; 4 Research Center for Agrometeorology, Viet Nam Institute of Meteorology, Hydrology And Climate Change, Hanoi, Vietnam; 5 CGIAR Research Program on Climate Change, Agriculture and Food Security (CCAFS), International Rice Research Institute (IRRI), Hanoi, Vietnam; Sam Houston State University, UNITED STATES

## Abstract

The Mekong River Delta is the rice production hub in South-east Asia and has a key role in determining rice prices in the world market. The increasing variability in the local climate due to global climate changes and the increasing severity of the ENSO phenomenon threatens rice production in the region, which has consequences for local and global food security. Though existing mapping efforts delineate the consequences of saline water intrusion during El Niño and flooding events during La Niña in the basin, research to predict future impacts in rice production is rather limited. The current work uses ORYZA, an ecophysiological model, combined with historical climate data, climate change scenarios RCP4.5 and 8.5 and climate-related risk maps to project the aggregate productivity and rice production impacts by the year 2050. Results show that in years of average salinity intrusion and flooding, the winter-spring rice crop in the MRD would experience an average annual decrease of 720,450 tons for 2020–2050 under the RCP4.5 scenario compared to the baseline of 2005–2016 average and another 1.17 million tons under the RCP8.5 scenario. The autumn-winter crop would decrease by 331,480 tons under RCP4.5 and 462,720 tons under RCP8.5. In years of severe salinity intrusion and flooding, the winter-spring rice crop would decrease by 2.13 million tons (10.29% lower than the projection for an average year) under RCP4.5 and 2.5 million tons (13.62%) under RCP8.5. Under severe conditions, the autumn-winter crop would have an average decrease of 1.3 million tons (7.36%) under RCP4.5 and 1.4 million tons (10.88%) for the RCP8.5 scenario. Given that most of the rice produced in this area is exported, a decline in rice supply at this scale would likely have implications on the global market price of rice affecting global food security. Such decline will also have implications for the rural economy and food security of Vietnam. Suggestions for corrective measures to reduce the impacts are briefly discussed.

## 1. Introduction

**Fig 1:**
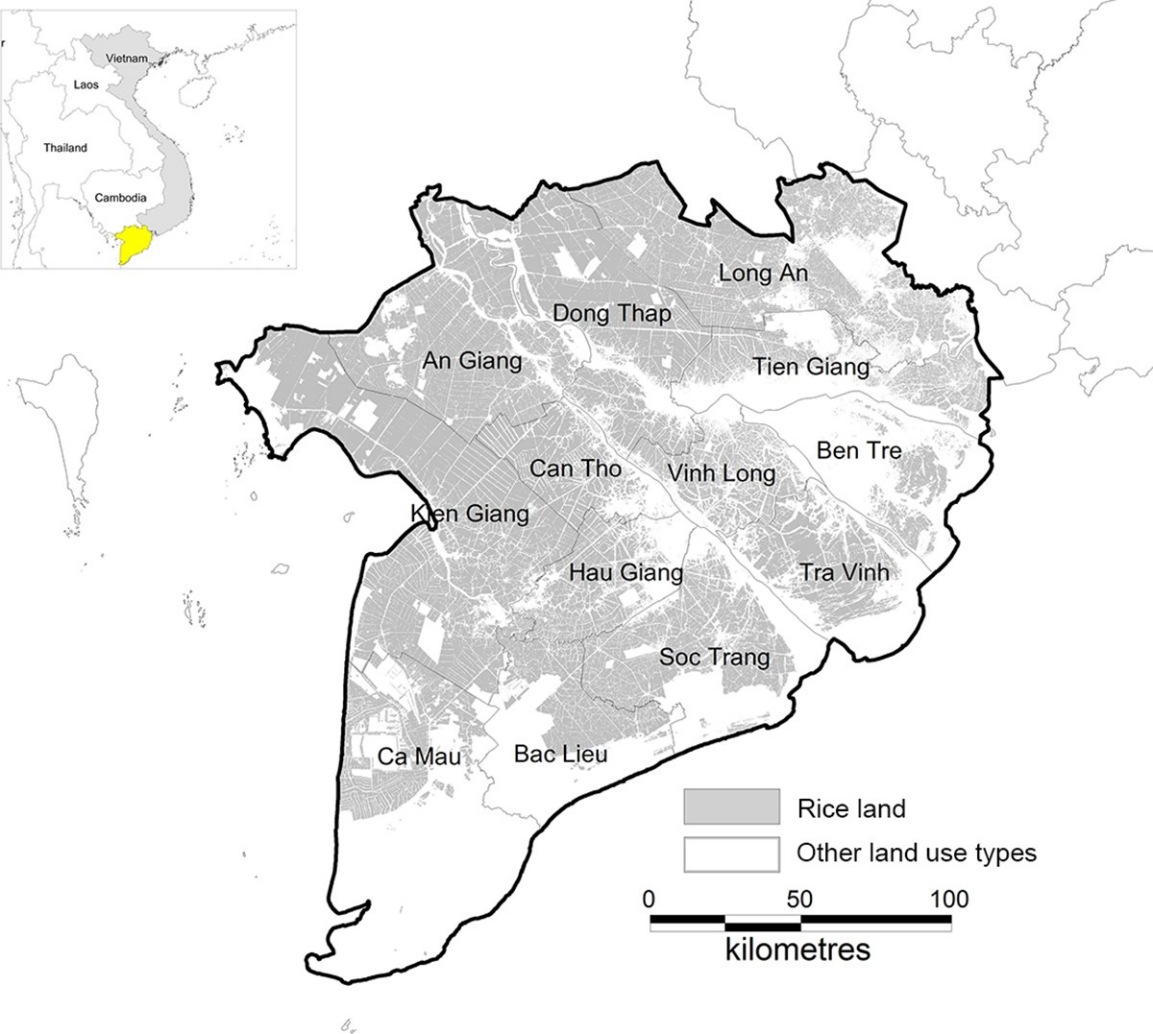
Location of the Mekong River Delta region in the map of Vietnam (yellow area in small map) and distribution of the region’s rice land in 2014.

The Mekong River Delta (MRD) (Fig 1) is the *rice bowl* of Vietnam, accounting for 55% of national rice production and 90% of the national rice exports valued at 2.85 billion USD in 2015 [1]. In 2017, the country produced 42.8 million tons of rice and contributed 7.5% of total global rice exports [2]. Any disruption to production in major export nations like Vietnam could create volatility in world markets, thereby affecting food security of rice importing nations [3]. A major threat is the El Niño–Southern Oscillation (ENSO) phenomenon, creating large inter-annual variations in precipitation ranging from severe drought to large-scale floods [4, 5]. ENSO is an ocean (surface temperature) and atmospheric (air pressure) phenomenon that influences climate and hydrology at a global scale [5]. The 2015–2016 El Niño, a warm ENSO phase, damaged nearly 250,000 ha of rice [6] in the MRD due to drought and saline intrusion. Severe floods coincide with the cold ENSO phase (i.e. La Niña years 2000, 2001, 2002 and 2005) which impacted 100,000 ha in 2000 due to the inundation of over 2.5meters of water [7]. There is a general consensus that ENSO-induced hydrological anomalies are increasing in severity as a result of global climate change [5].

ENSO events have a significant impact on the precipitation, temperature, sunshine hours and evaporation regimes of the MRD [5, 8, 9]. El Niño years are characterised by higher temperatures, evaporation, and sunshine hours [5] while La Niña years are marked by significantly higher precipitation [10]. The most significant difference is noticed from December to April. The observed monthly meteorological data [11] shows that during El Niño years, the temperature was about 1°C higher, the number of sunshine hours increased by about 30 hours/month and the additional evaporation averaged 25mm/month, which all clearly indicate a risk of drought. Average monthly rainfall in La Niña years was 80mm higher than in normal years, which is indicative of flood risk. According to the monitoring done by the Southern Institute of Water Resources Research [12], the salinity concentration of some rivers in the MRD, such as Co Chien and Hau, increases gradually from January to the highest values recorded in April (13.7 ‰ in Co Chien river and 6.7 ‰ in Hau river). During El Niño years, salinity concentration is about 2–4‰ higher than the average. On the contrary, salinity concentration in La Niña years declined 1–3‰ compared to the average.

Fig 1 illustrates the distribution of rice land of the MRD in 2014. Rice can be grown in three cropping seasons in this region: the winter-spring season (WS) from November to February, the summer-autumn season (SA) from April to July and the autumn-winter season (AW) from August to November. According to Yen *et al*. [9], salinity intrusion often occurs during the WS season in 10 provinces (i.e., Long An, Vinh Long, Ca Mau, Bac Lieu, Tien Giang, Hau Giang, Soc Trang, Tra Vinh, Kien Giang and Ben Tre). Flooding often occurs during the AW season in 9 provinces (i.e., Long An, Dong Thap, Ca Mau, Can Tho, Tien Giang, An Giang, Vinh Long, Hau Giang and Kien Giang). Six provinces face both salinity intrusion and flooding risks yearly. However, these risks are rarely observed during the SA season in the MRD.

Under the threat of escalating ENSO anomalies, future losses to rice production from drought and salinity intrusion (El Niño) and flooding (La Niña) coupled with changes in the mean climate [13] need to be quantitatively assessed. This is to ensure that response measures are developed to achieve climate resilient and sustainable agricultural development in the region. Such measures will lead to enhanced food security at national and global levels [8, 14]. Though studies were conducted to map risks of flooding and salinity intrusion spatially and temporally [15, 16], the projection of these risks on future rice production in the MRD has yet to be done. The research presented here address this gap in the literature using a locally-calibrated ecophysiological model, ORYZA [17–19], which simulates the impacts of abiotic stress on rice growth and development merged with climate risk maps. This study does not look at sea level rise, a wide known result of climate change that directly influences rice production by reducing the area of cultivation land [20]. The research only focused on the estimation of changes in rice production caused by fluctuations in climatic factors, particularly rainfall and temperature.

## 2. Materials and methods

### 2.1. The methodological framework

**Fig 2:**
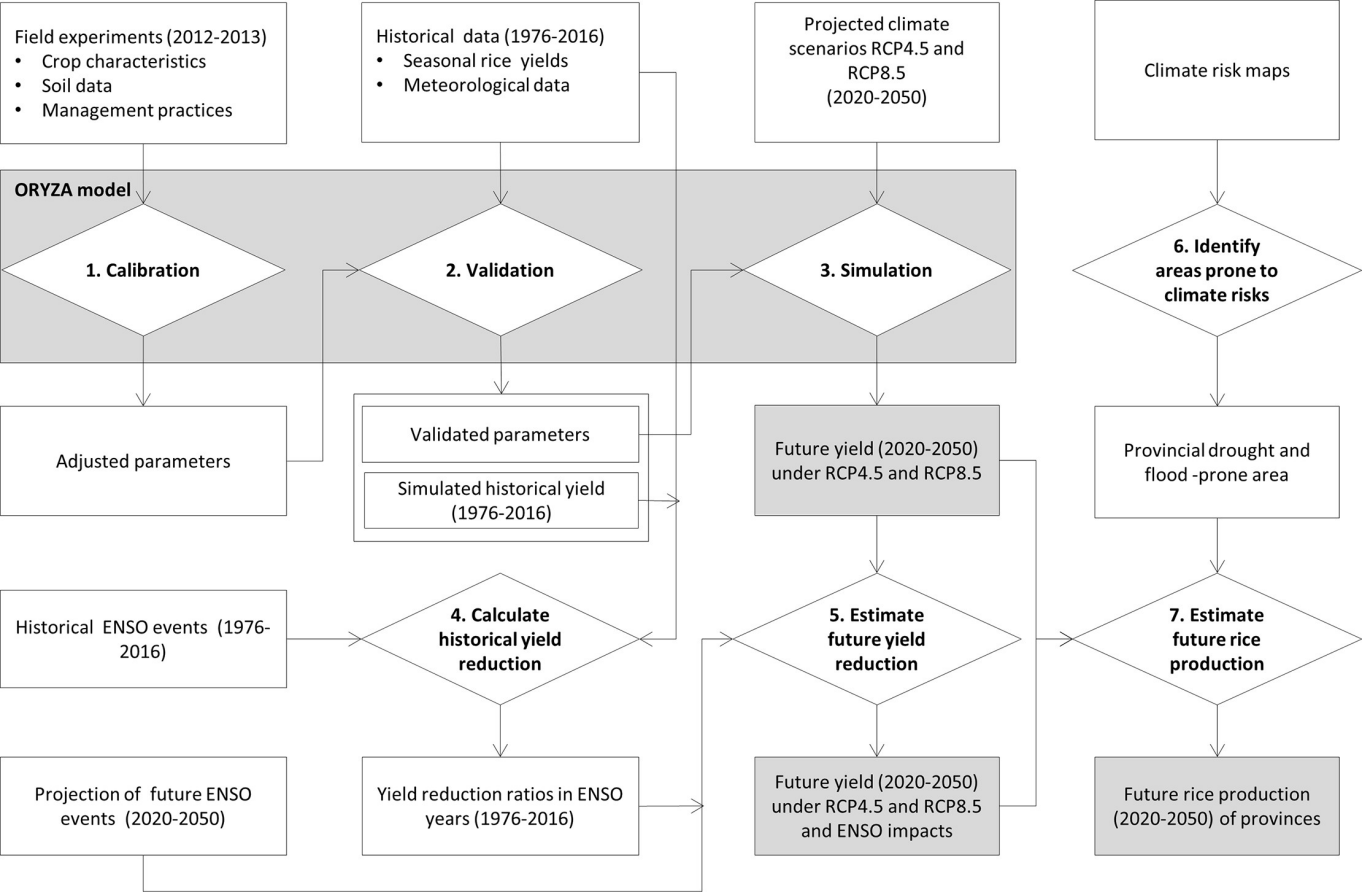
The methodological framework.

The methodological framework of this study is summarized in Fig 2, including two phases and seven steps as listed below:

Phase 1: Simulation of rice yield using ORYZA model

- Step 1: Model calibration. Field experimental data collected during the three crop seasons (winter-spring, summer-autumn and autumn-winter) in the period 2012–2013 were used as input data for the ORYZA model. The observed and simulated Leaf Area Index (LAI) and crop yield of each crop season were used to adjust the model’s parameters.
- Step 2: Model validation. Historic climatic data at the provincial level and the adjusted model parameters were used to simulate seasonal rice yield (ton/ha) of the 13 provinces of the MRD from 1976 to 2016. The best-fit between simulated and statistic yield was evaluated through an Efficiency Index.
- Step 3. Simulation of future rice yield. The validated model was then used to simulate future rice yield of each crop season from 2020 to 2050 under two projected climate scenarios RCP4.5 and RCP85 developed by the Ministry of Natural Resources and Environment (MoNRE) of Vietnam.

Phase 2: Analyse climatic impacts on rice yield and future rice production

- Step 4: Calculation of yield reduction ratios. The simulated yields for 1976–2016 obtained from step 2 were compared to observed yields for ENSO years to calculate the yield reduction amounts.
- Step 5. Estimation of future yield reduction. Projections of future ENSO events were applied to calculate future yield loss in high probability ENSO years using the yield reduction ratios calculated from step 4.
- Step 6. Identification of areas prone to climate risks. The climate risk maps were used to extract the area of rice lands of each province that are potentially affected by ENSO events.
- Step 7. Estimation of future rice production. Annual rice production per province in MRD for 2020–2050 were estimated using the simulated yields for 2020–2050 (step 3), the yield reduction ratios in high probability ENSO years (step 5), and the potentially affected rice areas (step 6).

Detailed description of these steps and corresponding input data are given in following sections.

### 2.2. Simulation of rice yield

In this study, the simulation of rice yield was done using ORYZA, a crop growth model developed by the International Rice Research Institute (IRRI). Among various crop models that can simulate rice yield, such as AquaCrop [21], WOFOST [22], SUCROS [23], SIMRIW [24], CERES-Rice [25], and DSSAT [26], the ORYZA model was specifically developed to simulate the growth and development of rice crop in lowland, upland, and aerobic rice ecosystems. The model was first introduced in 1994 and has been calibrated and validated for 18 popular rice varieties in 15 locations throughout Asia. ORYZA is an eco-physio-logical rice crop model with multiple modules for crop growth, evapo-transpiration, nitrogen dynamics, soil water balance, etc. [17, 27].

ORYZA is considered a comprehensive rice modelling tool applicable for different scenario analyses. Therefore, ORYZA was selected to simulate yields of major rice varieties in MRD under different climate scenarios. Detailed descriptions of the ORYZA model and its data requirement for model calibration are given by Wopereis *et al*. [18] and Li *et al*. [19].

#### 2.2.1. Model calibration (Step 1)

Calibration of ORYZA was done at plot level, using previous field experiment conducted during 2012–2013 in the MRD. The field experiments were established under a collaborative program between the Vietnam Institute of Meteorology, Hydrology and Climate change (IMHEN), Can Tho University (CTU), and International Rice Research Institute (IRRI) [28] [29], including 8 experimental plots in WS, 7 plots in SA and 4 plots in AW season. Eleven major local rice varieties developed by the Cuu Long Delta Rice Research Institute (i.e. OM8576, OM108-5, OM10636, etc.) and by Soc Trang province (i.e. ST5) [29] were used in this experiment. In general, the length of time from the establishing date to harvesting date of the rice varieties varies from 95 to 110 days. Field activities included monitoring of water levels in rice fields every two days; recording of fertilization schedules (date of fertilization, type and quantity of fertilizer); measuring rice density, plant height, tillering rate, growth status and recording of weed & pests infestation, etc. The leaf area index (LAI) was measured in four development stages: after transplanting, active tillering, panicle development, and flowering. Seasonal rice yields of each experimental plot were measured after harvest. Variables were used to adjust the model parameters for yield simulation under normal climate conditions (i.e. typical temporal distribution and average values of temperature and rainfall).

#### 2.2.2. Model validation (Step 2)

Performance of ORYZA with the adjusted parameters was validated at the provincial level, using seasonal rice yield and meteorological data observed from 1976 to 2016 [11], and soil data of provinces in the MRD. Simulation of rice yields was done for every season and every year in this time period. Provincial-level statistics of observed seasonal rice yields (YO, ton/ha) were provided for the period 1976–2016 by the General Statistics Office of Vietnam.

The meteorological data including air temperature (maximum, minimum and daily average); air humidity (minimum and daily average); daily rainfall; wind speed (daily average and maximum); and number of sunshine hours were collected from meteorological stations of the National Centre for Hydro-Meteorological Forecasting (NCHMF) of Vietnam. The observation network of the NCHMF includes 176 surface meteorological stations [30]. This study used historical meteorological data collected by the 11 stations located in the MRD [31].

The soil data used in this study was extracted from the existing soil map of the MRD at a scale of 1:500,000 [32] and soil maps of MRD’s provinces at a scale of 1:100,000 developed by the National Institute of Agriculture and Projection (NIAPP) [33]. The soil maps include 25 soil types classified following the soil classification system of Vietnam with detailed description of soil physical characteristics (e.g., parent materials, gleyic level, soil texture, and soil depth) and chemical characteristics (e.g., nitrogen, phosphorus, potassium, cation exchange capacity, etc.). In the MRD, the major soil types are acid-sulphate soil, alluvial soil, and saline soil, which cover 41%, 30%, and 19% of the total area, respectively. All other soil types such as sandy soil, degraded soils, yellowish—red soil, and peat soil only cover the remaining 10%. In this study, characteristics of alluvial and lightly saline soils, the two major soil types of MRD, were used to validate the ORYZA simulation.

The model efficiency (*E*_*f*_) was used to evaluate the best-fit of the simulated yield (YS, ton/ha) generated by ORYZA and the observed yield (YO, ton/ha) of each MRD’s province for the period 1976–2016. *E*_*f*_ value ranges from 1 to −∞. An efficiency value of 1 shows a perfect relationship between simulation and observation; a value of 0 occurs when an equal performance relative to the reference value; and a negative value indicates that the model predictions are worse than the observation average. *E*_*f*_ was calculated as:

$$E_{f}=1-\frac{\sum_{i=1}^{n}{\left(YO_{i}-YS_{i}\right)}^{2}}{\sum_{i=1}^{n}{\left(YO_{i}-\bar{YO}\right)}^{2}}$$ (Eq 1)

Where, n is the number of simulated years; YOi and YSi are observed and simulated yield (ton/ha) at year ith, respectively; $\bar{YO}$ is the average observed yield from 1976 to 2016.

#### 2.2.3. Simulation of future rice yield (Step 3)

The validated model was applied to estimate the rice yield of each crop season from 2020 to 2050 of 13 MRD provinces under projected climate scenarios. The four climate scenarios projected by MoNRE [20] consist of: low GHG concentration—RCP2.6; average GHG concentration—RCP4.5; relatively high GHG concentration—RCP6.0; and high GHG concentration—RCP8.5. These scenarios were developed based on adjustments to five regional climate change models, including AGCM/MRI [34], Providing Regional Climates for Impacts Studies (PRECIS) [35], Conformal Cubic Atmospheric Model (CCAM) [36], Regional Climate Model (RegCM) [37], and Weather Research and Forecast (clWRF) [38], and based on the global climate predictions described in the IPCC’s Fifth Assessment Report [39]. Accordingly, the regional climate change models were calibrated with temperature and rainfall data and extreme climate events (typhoon, hot, and cold spells) observed at 150 hydro-meteorological stations in Vietnam. After validating these models, MoNRE used projected temperature from all five models and projected rainfall from PRECIS to develop the final climate change scenarios for Vietnam.

This study only used the projected climate data of the two scenarios RCP4.5 and RCP8.5. By 2050, average annual temperature of the MRD is projected to increase by 1.4°C for the scenario RCP4.5 and 1.8–1.9°C for the scenario RCP8.5. Annual rainfall is predicted to increase 5–15% in the whole country. Annual rainfall in the MRD is estimated to increase by an average of 13% for the scenario RCP4.5, with a predicted rainfall increase from 5.8% in Ca Mau province to an 18.2% in Ben Tre province. The RCP8.5 scenario forecasts annual rainfall in the region to increase as much as 10.8% in Ca Mau province to 18.3% in Can Tho province.

MoNRE climate models estimate that the number of hot days (maximum daily temperature is greater than 35°C) may be an increasing trend across the entire country until 2100. Low rainfall in the dry season combined with high temperatures will lead to more severe drought in the future. The two climate scenarios RCP4.5 and RCP8.5 were used as inputs for the ORYZA model to estimate future seasonal rice yield (YF, ton/ha) from 2020 to 2050, assuming that farming conditions (i.e., infrastructure, cultivation area, rice varieties, fertilizers, farming techniques, soil, and irrigation condition) will not differ from those observed in the period 2005–2016.

### 2.3. Analysis of climatic impacts on rice production

#### 2.3.1. Calculate historical yield reduction (Step 4)

Because estimation of crop yield under extreme climate event was not included in ORYZA model, the simulated yields for WS, SA, and AW season in La Niña or El Niño years during the period 1976–2016 were compared to observed yields to calculate the yield reduction amounts. The seasonal yield reduction (D, %) caused by flooding during the AW season in La Niña years, and by salinity intrusion during the WS season in El Niño years was classified into 3 levels (l): high reduction (l = 1), moderate reduction (l = 2) and low reduction (l = 3). Because there is no flood or salinity intrusion risk during SA season (s = 2), D was considered to be equal zero (0). The yield reduction is estimated in the Eq 2.

$$D_{s}=\frac{{YO}_{s}-{YS}_{s}}{{YO}_{s}}*100$$ (Eq 2)

Where D_s_(%) is yield reduction ratios estimated per cropping season s; YO_s_ and YS_s_ are observed and simulated rice yields (ton/ha) per cropping season s, respectively.

#### 2.3.2. Estimation of future yield reduction (Step 5)

In this study, it is assumed that the yield reduction ratios will be the same in future El Niño and La Niña years. The predictions made by Zhang *et al*. [4] and Fer *et al*. [40] were used to identify the possibility, frequency, and intensity of likely El Niño and La Niña events in the future. The historical yield reduction calculated in the previous step was used to estimate seasonal rice yield reduction of 13 MRD provinces in the future El Niño and La Niña years for the period 2020–2050. According to these predictions, there are high possibilities to experience El Niño impacts during the WS seasons of 2020–2021, 2025–2026, 2026–2027, 2032–2033, 2035–2036, 2037–2038, 2038–2039, 2042–2043 and 2046–2047; and La Niña during the AW seasons of 2023, 2027, 2028, 2031, 2034, 2035, 2037, 2040, 2041, 2045, 2048, 2049, and 2050. In these high probability ENSO years, simulated seasonal rice yield under RCP4.5 and RCP8.5 (YS, ton/ha) is recalculated using the estimated reduction ratios D_s_ (%) to produce estimated future rice yield (YF, ton/ha). Due to provincial and seasonal differences in physical conditions (i.e., soil and irrigation) and management practices (i.e., fertilization, weeding, and sowing/transplanting), the estimated yield reduction levels (l) by season (s) will also be different. Therefore, levels of D were calculated for individual MRD provinces following Eq 3.

$${YF}_{sl}={YS}_{sl}*(1-D_{sl})$$ (Eq 3)

#### 2.3.3. Identification of areas prone to climate risks (Step 6)

Area (S, ha) of rice lands that will potentially be affected by salinity intrusion in future El Niño years and by floods in future La Niña years was estimated from the climate-related risks maps of MRD’s provinces. The maps were generated under collaboration of the Department of Crop Production (DCP) of the Ministry of Agriculture and Rural Development (MARD) of Vietnam, and the CGIAR Research Program on Climate Change, Agriculture and Food Security in Southeast Asia (CCAFS SEA) [9]. The maps clearly show the area and distribution of three risk levels (high, medium, and low) corresponding to three levels of potential yield loss (>70%, 30–70%, and <30%), respectively. The level and location of flood and salinity intrusion risks in future normal years and ENSO years were determined by provincial officials using available data (i.e. topography, and hydrology), infrastructures (i.e. dikes, road, and canals), current management practices, and local expert knowledge. Table 1 shows the percentage of potentially affected rice land by province extracted from these climate-related risks maps.

**Table 1:** Percentage of rice land potentially affected by salinity intrusion in El Niño years and flooding in La Niña years.

	Percentage of potentially affected rice land (%)							
	Salinity intrusion risk				Flooding risk			
Province	High	Medium	Low	No risk	High	Medium	Low	No risk
Long An	15.4	11.7	21.2	51.7	16.2	60.9	-	22.9
Tien Giang	2.1	0.2	47.3	50.4	26.8	16.2	2.0	55.0
Ben Tre	87.8	0.6	-	11.6	-	-	-	100.0
Tra Vinh	53.4	28.9	16.9	0.9	-	-	0.1	99.9
Vinh Long	8.1	20.5	28.3	43.1	-	38.4	28.3	33.2
Dong Thap	-	-	-	100.0	47.9	41.6	3.8	6.8
An Giang	-	-	11.1	88.9	55.2	15.1	0.0	29.7
Kien Giang	25.9	0.4	0.0	73.8	0.0	28.2	66.7	5.1
Can Tho	-	-	0.0	100.0	27.2	29.4	16.9	26.5
Hau Giang	45.8	4.1	10.6	39.5	59.2	8.9	31.8	-
Soc Trang	27.1	39.3	0.7	32.9	-	-	-	100.0
Bac Lieu	10.7	19.0	48.3	22.0	-	-	-	100.0
Ca Mau	75.9	2.4	-	21.7	-	25.3	-	74.7

In this study, three levels of rice yield reduction under flood and salinity intrusion conditions in ENSO years are considered to be consistent with the three risk levels (i.e. high, medium and low) defined in the climate-related risk maps.

#### 2.3.4. Estimation of future rice production (Step 7)

Following Eqs 4 and 5, the seasonal (PPs, ton) and annual (PPa, ton) rice production of a province for the period 2020–2050 was calculated from future simulated yield (YF, ton) and potential affected area (S, ha) extracted from the climate-related risks maps. It is noted that number of rice seasons (s) per year and risk level (l) varies from province to province.

$${\mathrm{PP}}_{s}=\sum_{l=1}^{3}\left({\mathrm{YF}}_{\mathrm{sl}}*S_{\mathrm{sl}}\right)$$ (Eq 4)

$${\mathrm{PP}}_{a}=\sum_{s=1}^{3}{\mathrm{PP}}_{s}$$ (Eq 5)

## 3. Results and discussion

### 3.1. Simulation of rice yield

#### 3.1.1. Calibration of the ORYZA model

**Fig 3:**
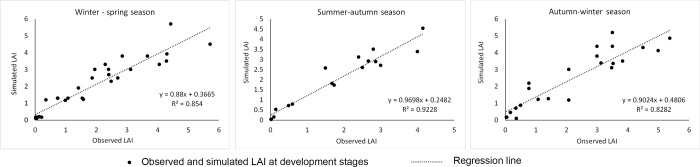
Relationship between observed Leaf Area Indexes (LAI) from experimental plots in Soc Trang province for winter-spring, summer-autumn, and autumn-winter season during 2012–2013, and simulated indexes generated by the ORYZA model.

Parameters of the ORYZA model were re-calibrated using fields experiments in the WS, SA and AW seasons during 2012–2013. Fig 3 shows the relationship between leaf area indexes (LAI) measured in experimental plots (Observed LAI) and the indexes simulated by the calibrated ORYZA model. The result shows that observed and simulated LAI are statistically indistinguishable. The coefficient of determination (R^2^) of the two variables range from 0.82 to 0.92. This proves that the ORYZA model performed well with the adjusted parameters and the results were statisticaly significant.

Table 2 shows the relationship between observed (YO, ton/ha) and simulated rice yields (YS, ton/ha) of experimental plots in WS, SA, and AW seasons during the period of 2012–2013. The high value of coefficient of determination (R^2^), which varies from 0.78 (for AW) to 0.96 (for SA), demonstrates that YS is highly correlated to YO. Furthermore, the small seasonal value of the Root Mean Square Error (RMSE), which ranges from 0.35 to 0.47, proves that YO and YS are close to the regression line of the two variables.

**Table 2:** Comparison between observed (YO, ton/ha) and simulated (YS, ton/ha) rice yields of experimental plots implemented in Soc Trang province for winter-spring, summer-autumn, and autumn-winter season during 2012–2013.

	Seasonal rice yield, ton/ha*								
Plot	Winter-spring			Summer-autumn			Autumn-winter		
	YS	YO	YO-YS	YS	YO	YO-YS	YS	YO	YO-YS
1	6.18	6.52	0.35	4.30	4.21	-0.09	5.29	5.04	-0.25
2	5.12	5.00	-0.12	3.81	3.21	-0.60	5.62	5.69	0.07
3	5.61	5.03	-0.58	5.78	5.42	-0.36	5.45	5.48	0.03
4	6.18	5.59	-0.60	-	-	-	6.44	5.39	-1.05
5	6.38	6.40	0.02	-	-	-	6.37	5.87	-0.50
6	6.01	6.07	0.06	5.87	5.78	-0.08	5.08	4.78	-0.30
7	7.42	7.82	0.40	-	-	-	7.31	7.21	-0.10
8	6.71	6.65	-0.06	-	-	-	-	-	-
RMSE	0.35			0.36			0.47		
R^2^	0.89			0.96			0.78		

“-”: no field experiment

#### 3.1.2. Validation of the ORYZA model

**Fig 4:**
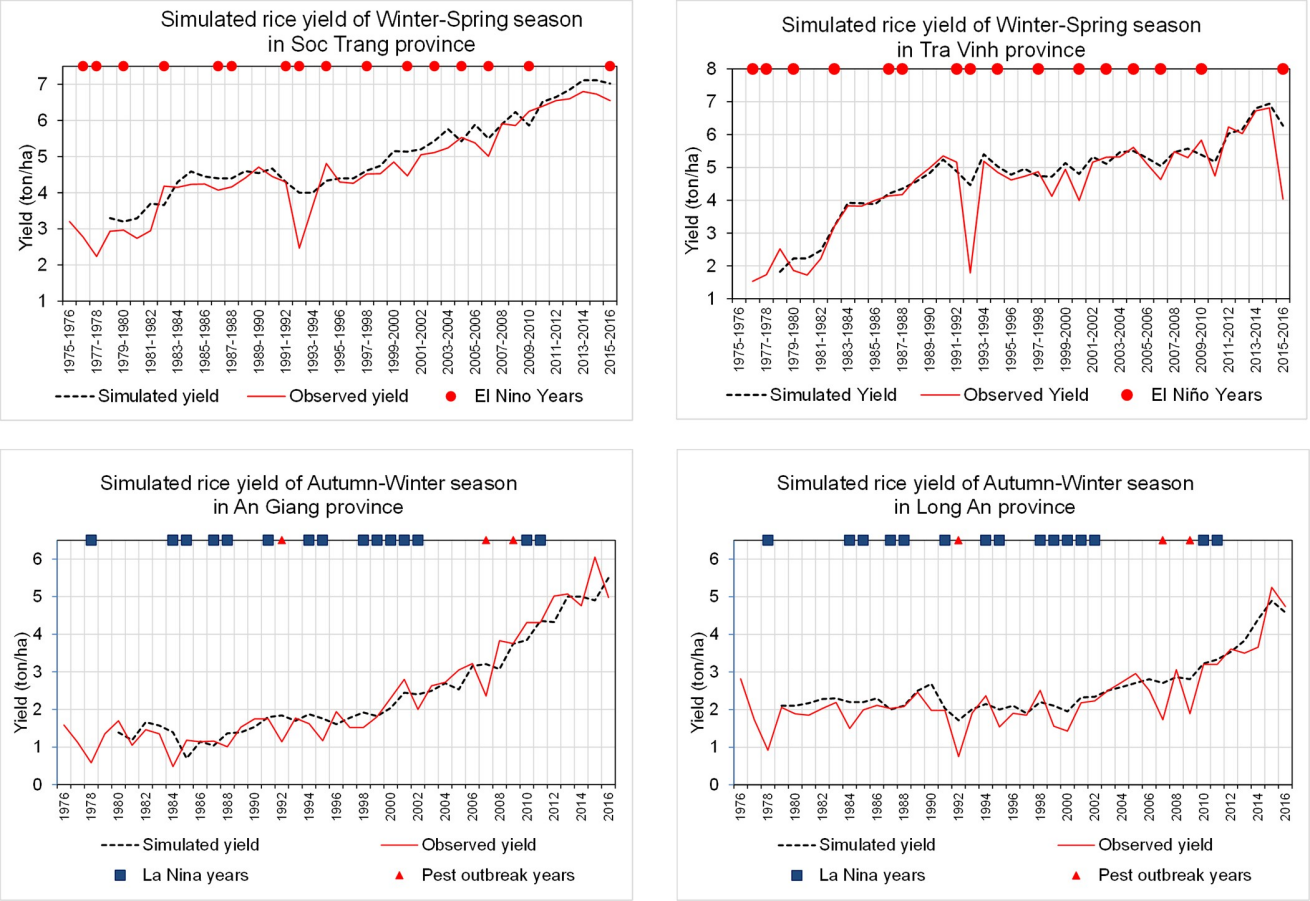
Simulated and observed rice yield of WS season in (a) Soc Trang and (b) Tra Vinh provinces, and of AW season in (c) An Giang and (d) Long An provinces for the period 1976–2016.

The rice yield of each of the MRD provinces during the period 1976–2016 was simulated given the adjusted sets of ORYZA parameters. Fig 4 presents the comparison between the simulated and observed rice yield of Soc Trang and Tra Vinh provinces in the WS cropping season, and An Giang and Long An provinces in the AW cropping season. The figures illustrate that simulated yields are quite close to the observed yields in both cropping seasons. The observed and simulated rice yields by cropping season and by province from 1976 to 2016 are shown in S1 File.

Interestingly, during ENSO years (red circles in Fig 4A and 4B for El Niño years, and blue squares in Fig 4C and 4D for La Niña years), simulated yields are generally higher than observed yields. This is because the extremely unfavorable conditions for rice growth (i.e. salinity intrusion and drought) are not included in the ORYZA estimations. Furthermore, the observed rice yields of AW seasons in 1992, 2002 and 2009 are far below the simulated yields although there was no La Niña effect during these years. These declines of rice yield can be explained by serious outbreaks of the brown plant hopper and the rice grassy stunt virus (red triangles in Fig 4C and 4D) that occurred during these years and significantly damaged rice production in the western provinces including An Giang, Long An, Dong Thap, Tien Giang, etc. Although ORYZA is considered a comprehensive rice modeling tool applicable for different scenario analyses [17, 41], estimations of rice yield under such extreme conditions need to be done separately outside of model simulation.

Despite some differences in rice yield estimation, the good fit confirms that the recalibrated ORYZA model can be used to estimate rice yields in the MRD. The best-fit of simulated seasonal rice yields of provinces for the period 1976–2016 is proved by the coefficient for model efficiency (E_f_) (Table 3), which ranges from 0.70 to 0.97 for the WS and 0.80 to 0.96 for the AW season.

**Table 3:** The coefficient of determination (Ef) calculated for validation of seasonal yield estimation for the period 1976–2016 of 13 MRD provinces.

	Model efficient coefficient (Ef) by province**												
Cropping season*	Long An	Tien Giang	Ben Tre	Tra Vinh	Vinh Long	An Giang	Kien Giang	Hau Giang	Soc Trang	Bac Lieu	Ca Mau	Can Tho	Dong Thap
WS	0.97	0.96	0.70	0.75	0.95	0.88	0.95	0.80	0.86	0.94	-	-	-
AW	0.90	0.81	-	-	0.92	0.96	0.94	0.82	-	-	0.80	0.87	0.85

* WS = Winter-Spring; AW = Autumn-Winter cropping season

** The provinces with no Ef value (-): the cropping season is not applicable or observed data series is too short to evaluate the estimation.

### 3.2. Impacts of flooding and salinity intrusion on rice yields

The variation of historical rice yields of the WS season (Fig 4A and 4B) shows that there were significant declines due to El Niño impacts in the coastal provinces such as Soc Trang and Tra Vinh. The instances of unusual low yield in WS season in 1992–1993 and 2015–2016 implies severe negative effects of droughts and salinity intrusion.

**Fig 5:**
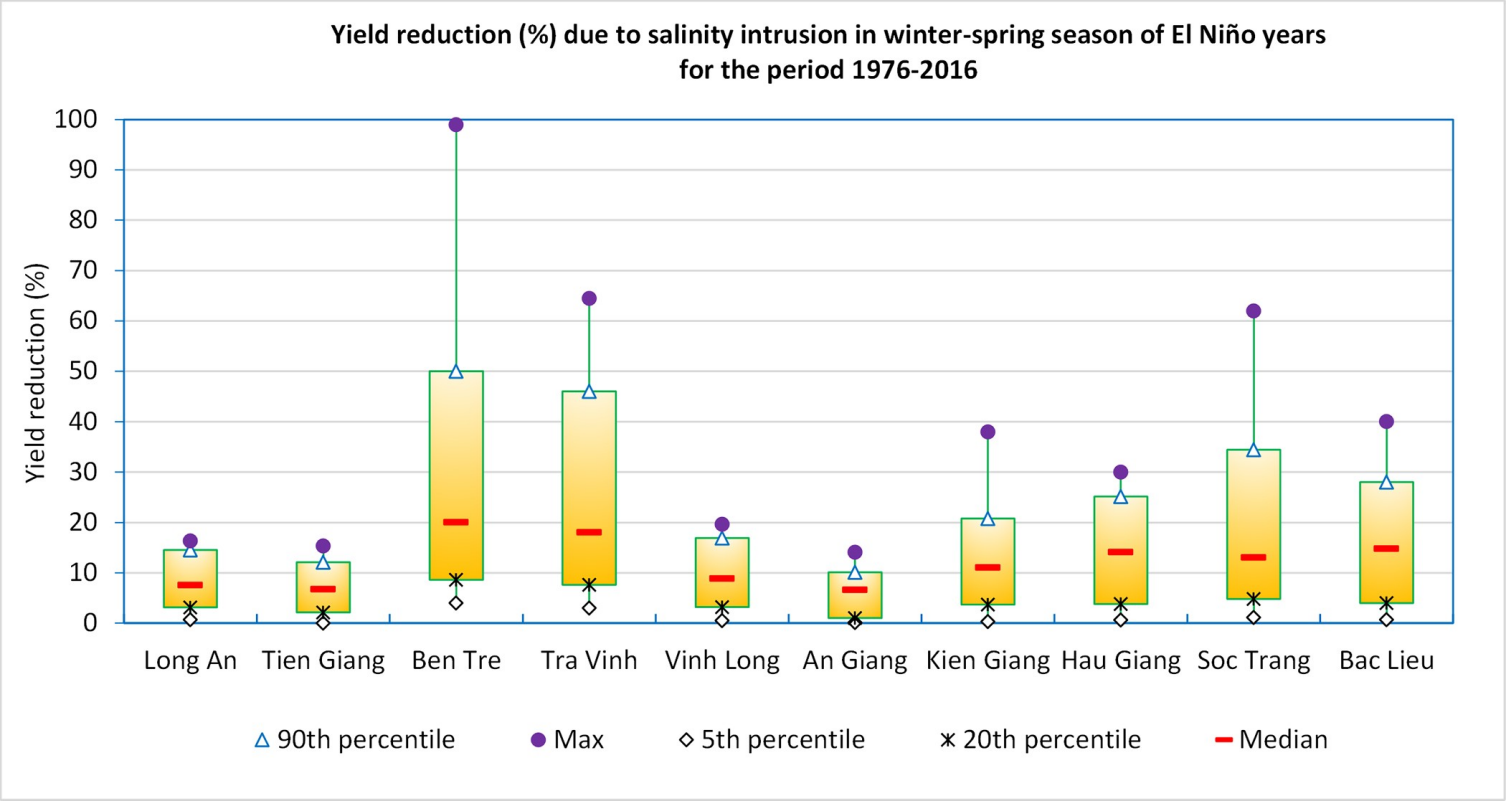
Yield reduction (%) due to salinity intrusion in winter-spring season of El Niño years for the period 1976–2016.

Fig 5 shows the cumulative probability of yield reduction due to salinity intrusion in WS season in El Niño years. There is a large difference in yield reduction among the MRD provinces. Ben Tre and Tra Vinh provinces record the highest reductions of 99% and 65%, respectively. Less affected provinces such as Long An, Tien Giang and Vinh Long show the reduction of less than 20%.

**Fig 6:**
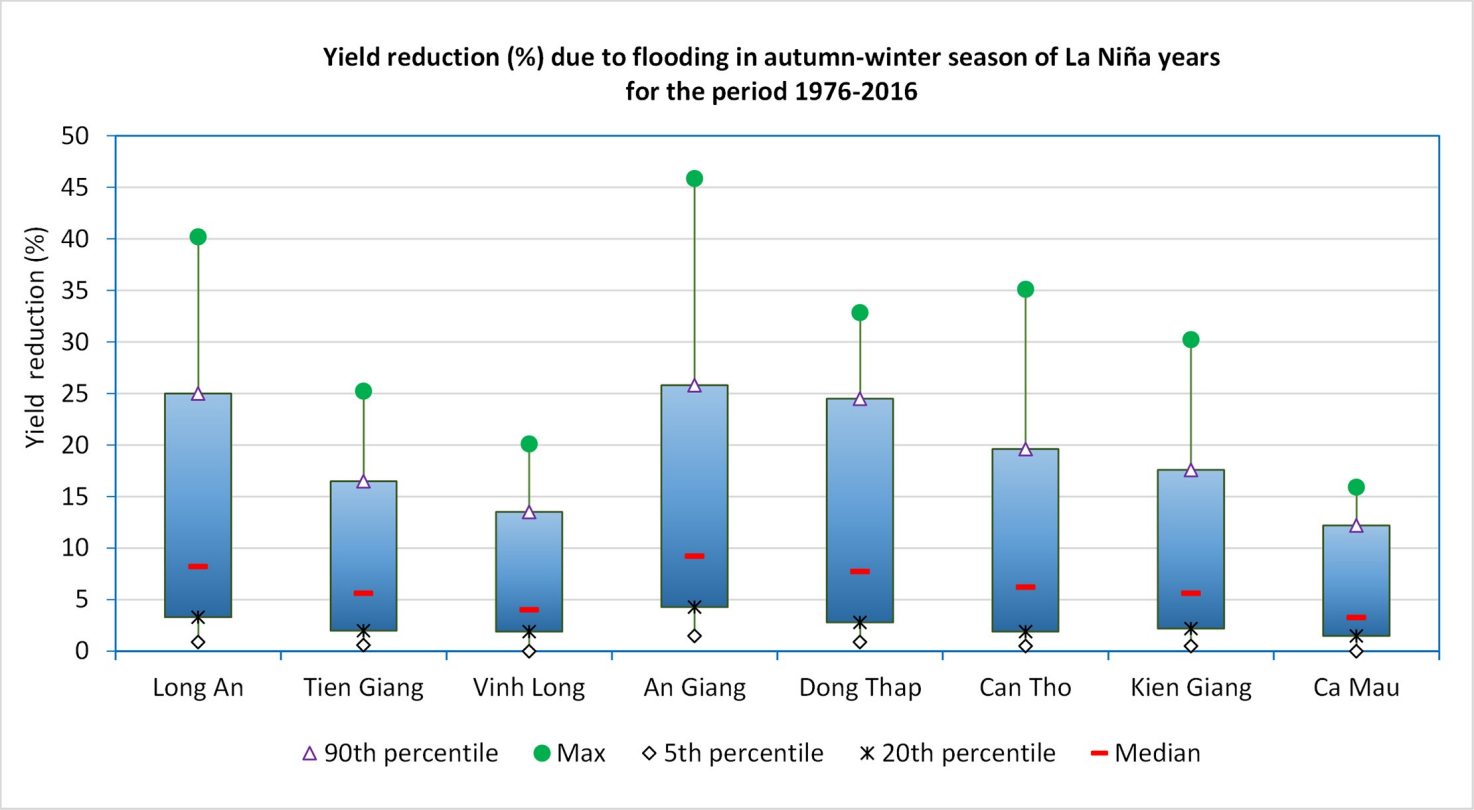
Yield reduction (%) due to flooding in autumn-winter season of La Niña years for the period 1976–2016.

During the past La Niña years such as 1984 and 1992, rice yields in AW season in low elevation parts of the MRD (e.g., An Giang, Dong Thap and north of Long An) (Fig 4C and 4D) also reduced. Because of higher rainfall than the long–term average in La Niña years, unusually severe flooding occurred and damaged AW rice from September to November. In addition, the high humidity in La Niña years also favored growth and outbreaks of pests and diseases and, therefore, reduced rice yield. Generally, the yield reduction in La Niña years has significantly lower affect than that in El Niño years. The largest reduction was in An Giang (about 45%), and the smallest reduction was in Ca Mau (about 15%) for the most severe year (Fig 6).

Based on the calculated yield reduction of the each affected provinces (Figs 5 and 6) from 1976 to 2016, the reduction ratio was divided into 3 levels: low, moderate, and high. Major differences are observed in the yield reductions between provinces due to the differences in local adaptation capacities (e.g. infrastructure, soil environment, irrigation, and management practices); therefore, the reduction levels were calculated for each of the MRD provinces. Values of yield reduction levels are presented in Table 4, which provides the basis for calculating the yield decline in ENSO years for the period 2020–2050.

**Table 4:** Levels of rice yield reduction ratio of MRD’s provinces due to negative impacts of El Niño in WS season and La Niña in AW season.

	Yield reduction ratio (D, %) by province												
Level	Long An	Tien Giang	Ben Tre	Tra Vinh	Vinh Long	An Giang	Kien Giang	Hau Giang	Soc Trang	Bac Lieu	Ca Mau	Can Tho	Dong Thap
El Niño years													
*High*	≥ 15	≥ 12	≥ 50	≥ 46	≥ 17	≥10	≥ 21	≥ 25	≥ 34	≥ 28	-	-	-
*Medium*	4–14	3–11	10–49	9–45	4–16	2–9	5–20	5–24	6–33	5–27	-	-	-
*Low*	≤ 3	≤ 2	≤ 9	≤ 8	≤ 3	≤ 1	≤ 4	≤ 4	≤ 5	≤ 4	-	-	-
La Niña years													
*High*	≥ 25	≥ 17	-	-	≥ 14	≥ 26	≥ 18	-	-	-	≥ 12	≥ 20	≥ 25
*Medium*	4–24	3–16	-	-	3–13	5–25	3–17	-	-	-	3–11	3–19	4–24
*Low*	≤ 3	≤ 2	-	-	≤ 2	≤ 4	≤ 2	-	-	-	≤ 2	≤ 2	≤ 3

### 3.3. Estimation of future rice yield

#### 3.3.1. Estimation of rice yield under climate change scenarios

The future seasonal rice yield of each province in the MRD was estimated for the 2020–2050 period based on the projected climate pattern under the two climate change scenarios RCP4.5 and RCP8.5 [20]. Estimated rice yields of MRD provinces in the WS, SA and AW seasons from 2020 to 2050 are given in S2 File. The average seasonal rice yield of this period was compared with the average yield of the last 10 years (2005–2016) to analyze the impact of climate change. In this estimation, impacts of flooding and salinity intrusion were not taken into consideration.

Data presented in Table 5 shows that the future rice yield may reduce by an average of 1.93% in the WS, 1.74% in SA and 1.66% in the AW season under the average GHGs concentration scenario (RCP4.5) compared to the 10-year-average of present rice yield (2005–2016). The yield reduction ratios will be highest (>2%) in Soc Trang, Bac Lieu and Dong Thap provinces for WS, Tien Giang and Dong Thap provinces for SA, and in Soc Trang, Bac Lieu and An Giang provinces for AW season.

**Table 5:** Differences between average future (2020–2050) and present (2005–2016) rice yields in the winter-spring (WS), summer-autumn (SA) and autumn-winter (AW) cropping seasons.

Province	Scenario*	Average seasonal rice yield (ton/ha) **								
		WS			SA			AW		
		Present	Future	D (%)	Present	Future	D (%)	Present	Future	D (%)
Long An	RCP4.5	5.86	5.78	1.37	4.19	4.12	1.67	3.28	3.22	1.83
	RCP8.5		5.46	6.83		4.00	4.53		3.12	4.88
Tien Giang	RCP4.5	6.71	6.58	1.94	4.94	4.84	2.02	4.42	4.35	1.58
	RCP8.5		6.28	6.41		4.71	4.66		4.2	4.98
Ben Tre	RCP4.5	4.99	4.89	2.00	4.11	4.04	1.70	4.16	4.09	1.68
	RCP8.5		4.67	6.41		3.94	4.14		3.98	4.33
Tra Vinh	RCP4.5	5.54	5.44	1.81	4.84	4.77	1.45	4.67	4.61	1.28
	RCP8.5		5.18	6.50		4.62	4.55		4.46	4.50
Vinh Long	RCP4.5	6.58	6.48	1.52	4.75	4.68	1.47	4.43	4.37	1.35
	RCP8.5		6.17	6.23		4.56	4.00		4.24	4.29
Dong Thap	RCP4.5	6.99	6.83	2.29	5.23	5.12	2.10	4.32	4.26	1.39
	RCP8.5		6.53	6.58		5.01	4.21		4.09	5.32
An Giang	RCP4.5	7.29	7.15	1.92	4.84	4.75	1.86	4.46	4.37	2.02
	RCP8.5		6.82	6.45		4.58	5.37		4.19	6.05
Kien Giang	RCP4.5	6.68	6.53	2.25	4.86	4.77	1.85	4.07	4.01	1.47
	RCP8.5		6.23	6.74		4.62	4.94		3.87	4.91
Can Tho	RCP4.5	7.06	6.93	1.84	4.70	4.62	1.70	3.89	3.83	1.54
	RCP8.5		6.64	5.95		4.51	4.04		3.72	4.37
Hau Giang	RCP4.5	6.69	6.58	1.64	4.53	4.46	1.55	4.46	4.38	1.79
	RCP8.5		6.30	5.83		4.36	3.75		4.29	3.81
Soc Trang	RCP4.5	6.13	5.98	2.45	4.84	4.75	1.86	4.46	4.37	2.02
	RCP8.5		5.75	6.20		4.58	5.37		4.22	5.38
Bac Lieu	RCP4.5	6.27	6.14	2.07	5.10	5.01	1.76	4.82	4.72	2.07
	RCP8.5		5.87	6.38		4.90	3.92		4.60	4.56
Ca Mau	RCP4.5	-	-	-	4.18	4.11	1.67	3.76	3.70	1.60
	RCP8.5	-	-	-		3.98	4.78		3.59	4.52
Regional average	RCP4.5	6.40	6.28	1.93	4.70	4.62	1.74	4.25	4.18	1.66
	RCP8.5		5.99	6.38		4.49	4.48		4.04	4.76

* Climate change scenarios: RCP4.5 = average GHGs concentration; RCP8.5 = high GHGs concentration

** D = yield reduction ratio (%); (-) the cropping season is not applicable

Under the high GHGs concentration scenario (RCP8.5), the average reduction ratio is expected to be 6.38% in the WS, 4.48% in the SA and 4.76% in the AW season. Highest reduction ratio in all three cropping seasons will be in An Giang, which is one of the largest rice producing provinces in the MRD. These results are consistent to a study done by Jiang et al. [42], which predicted irrigated rice yield declines by 4.7% in WS season. Nevertheless, it should be noted that the study did not consider the impacts of ENSO events which we have reported here in the subsequent sections.

**Fig 7:**
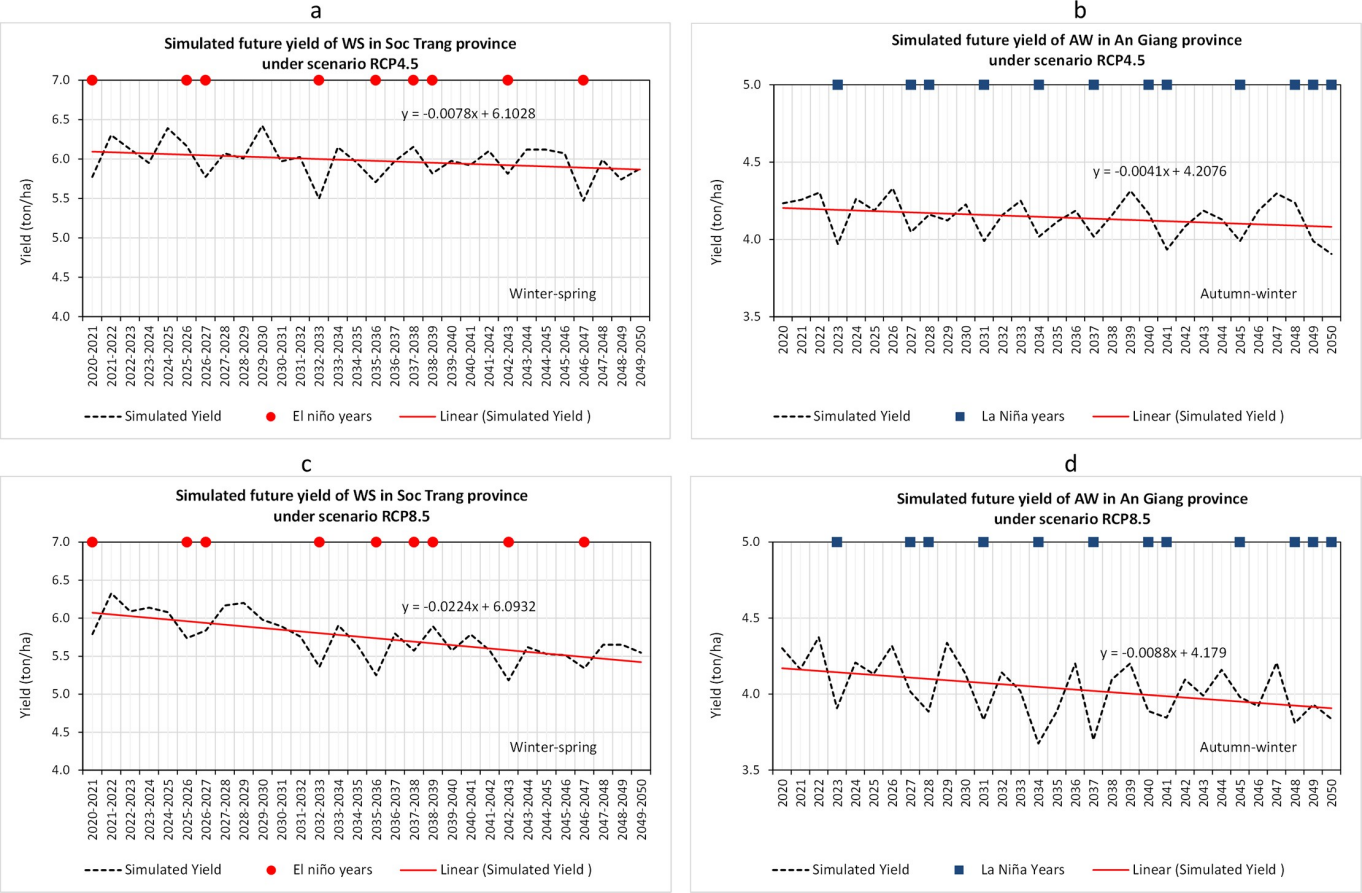
Simulated future rice yield of WS in Soc Trang province and AW in An Giang province from 2020 to 2050 under RCP4.5 and RCP8.5 climate change scenarios.

Fig 7 illustrates simulated rice yield of WS in Soc Trang province and AW in An Giang province under scenario RCP 4.5 (Fig 7A and 7B, respectively) and scenario RCP 8.5 (Fig 7C and 7D, respectively). By 2050, the average rice yield of WS will likely drop (present average yield) to 5.5–6 ton/ha, depending on the level of GHG concentrations. Similarly, the average rice yield of AW will also drop to below 4 ton/ha.

In ENSO years, the rice yield is generally below the trend line, despite the fact that ENSO impacts are not explicitly considered in the simulations. This is reasonable, as climate change scenarios in Vietnam are developed based on dynamic downscaling [43] and inputs from regional climate change models which originated from the IPCC’s global climate change model products.

#### 3.3.2. Estimation of future rice yield and production in ENSO years

As presented in section 4.1, severe drought and salinity intrusion in El Niño years and unusually severe floods in La Niña years were the main reasons that lead to the decline in rice yield in the MRD in the past. With the future ENSO events [4, 40], rice production in the MRD will likely face even more severe declines from 2020 to 2050.

The yield reduction ratios calculated for the past ENSO years are used to estimate potential reduction of rice yield in the future. Accordingly, the rice yield under climate change scenarios that present higher incidences of severe conditions (calculated in section 4.3.1) will further decrease rice yield in future ENSO years. The severity of yield reduction depends on the level of seasonal yield reduction ratio calculated in the section 4.2. The estimated rice production of the MRD provinces based on the highest potential yield reduction ratios and climate-risk areas (Section 2.3.3) in the future (2020–2050) are presented in Table 6.

**Table 6:** Estimation of future rice production of MRD’s provinces under impacts of climate change scenarios and ENSO events.

Province	Rice production, 10^6^ ton						
	Present (2005–2016)	Future without ENSO		Future with ENSO			
		RCP 4.5	RCP 8.5	RCP 4.5		RCP 8.5	
				El Niño	La Niña	El Niño	La Niña
Long An	2.53	2.49	2.38	2.26	2.42	2.16	2.32
Tien Giang	1.18	1.16	1.12	1.14	1.12	1.10	1.08
Ben Tre	0.20	0.19	0.18	0.10	0.19	0.10	0.18
Tra Vinh	1.14	1.12	1.08	0.78	1.12	0.75	1.08
Vinh Long	0.93	0.92	0.88	0.87	0.90	0.84	0.87
Dong Thap	3.12	3.05	2.95	3.05	2.69	2.95	2.60
An Giang	3.75	3.68	3.53	3.68	3.19	3.53	3.06
Kien Giang	4.20	4.12	3.96	3.58	4.08	3.45	3.92
Can Tho	1.27	1.25	1.21	1.25	1.15	1.21	1.11
Hau Giang	1.12	1.10	1.07	0.86	0.96	0.83	0.93
Soc Trang	1.93	1.89	1.82	1.53	1.89	1.48	1.82
Bac Lieu	0.96	0.94	0.92	0.89	0.94	0.87	0.92
Ca Mau	0.46	0.45	0.44	0.45	0.44	0.44	0.43
MRD	22.79	22.37	21.52	20.45	21.12	19.69	20.32
Rice lost (%)		1.86	5.58	10.29	7.36	13.62	10.88

Data in Table 6 show that future annual rice production of the MRD may decrease by 1.86% (under RCP4.5) or 5.58% (under RCP8.5) compared to the average value recorded in recent years (2005–2016) due to changes in climate conditions without ENSO projections. In cases of severe drought and salinity intrusion in El Niño years, rice production in the WS season will be significantly damaged, especially in Ben Tre, Tra Vinh and Soc Trang provinces. This will lead to the loss of 10.29% and 13.62% of annual rice production under the RCP4.5 and RCP8.5 scenario, respectively. In cases of severe flooding in La Niña years, the annual rice production will also be affected. The large decline in AW rice production of Dong Thap, An Giang and Hau Giang provinces will bring annual production down by 7.36% (for RCP4.5) or 10.88% (for RCP8.5).

This study shows that climate change will lead to adverse impacts on rice production in the MRD, especially during ENSO years. Therefore, it is necessary to promote the research and selection of seedlings, breeding livestock and sea-products that use less fresh water, adapted to drought and saline conditions. On the other hand, production should be shifted, reducing the area of rice cultivation, and instead focusing on intercropping of rice-fish and rice-shrimp to reduce water use in agricultural production and adapt to the increasing scarcity of fresh water. For short-term adaptation, the current 6-month forecast of potential ENSO probability is quite high, so it is important to research and develop production plans to adjust the cropping systems appropriately with El Niño or La Niña scenarios. This study did not consider impacts on quality but a reduction in rice quality due to salinity can lead to lower market value and revenues. It can also be expected that changes in technology and management practices can possibly mitigate the impacts, which could not be included in the current projections. There is a need for further investigation in this direction.

## 4. Conclusions

The analysis of historical rice yield data from 1976–2016 confirms the significant impact of ENSO on rice yields in the MRD provinces. Previous El Niño years often lead to an increase in salinity intrusion in WS crops, for which rice yield could has been recorded to decrease by 65%, as seen in Tra Vinh in 1992/1993, and up to as much as 99% as observed in Ben Tre in 2015–2016. It is to be noted that a decline in 50% of the yield is unlikely to turn a profit under existing cost and price conditions. Such declines can lead farmers into debt traps as observed in many parts of South Asia (e.g., Myanmar, India). In contrast, previous La Niña years lead to increased flooding in AW season, which decreased rice yield as much as 46% as seen in An Giang in 1985. La Niña similarly impact rice yield and profitability, although the effects are generally less severe than El Niño years. In forecasted years of average salinity intrusion and flooding, WS rice crops in the MRD are projected to decrease by 720,450 tons during 2020 to 2050, under the RCP4.5 scenario and decrease by 1.17 million tons under the RCP8.5 scenario compared to 2005–2016 average. AW crop would decrease about 331,480 tons under RCP4.5 and about 462,720 tons under RCP8.5. In the case of severe salinity intrusion and flooding projections, the WS rice crop in the MRD would decrease by 2.13 million tons compared to the 2005–2016 average under RCP4.5 and by 2.5 million tons under RCP8.5. The AW crop would decrease about 1.3 million tons under RCP4.5 and 1.4 million tons for the RCP8.5 scenarios. These projected declines would also have implications on world market prices of rice and, hence, could affect food security of nations relying on rice imports, given the fact that most of the rice from this area is exported. Though RCP4.5 (with a projected mean global temperature increase of 1.8°C) is closer to the goal of the Paris Agreement to limit the warming well below 2°C by the end of the century, the projections show that there would be significant damage to rice production in the MRD. The impact differential between RCP8.5 and RCP4.5 projects the possible gains from shifting to a lower GHG concentration pathway and highlights the need to intensify global emission reduction efforts, as well as, the significant need to increase adaptive efforts at local scales. Adaptive actions, such as switching to salt/flooding tolerant or short duration rice varieties may help to reduce these impacts. Adjusting the planting dates and applying water saving measures (e.g., alternate wetting and drying practice) are also important management practices for rice production in the MRD. Lastly, converting rice monoculture farms to other integrated production systems such as rice-fish, rice- vegetables, fish-fish, etc. should be considered as options to avoid the economic repercussions of declines in yield.

## Supporting information

S1 File Observed and simulated rice yield (ton/ha) of MRD provinces in winter-spring and autum-winter seasons from 1976 to 2016. (XLSX)

S2 File Estimated rice yield (ton/ha) of MRD provinces in winter-spring, summer-autumn and autum-winter seasons from 2020 to 2050 under climate scenarios RCP4.5 and RCP8.5. (XLSX)
